# Role of MAPK Pathways in the Pathogenesis of Vitiligo

**DOI:** 10.3390/cimb48060546

**Published:** 2026-05-23

**Authors:** Yuexi Liu, Yukun Yuan, Xiaoyi Shi, Rongsi Sun, Xiaolan Ding

**Affiliations:** Department of Dermatology, Peking University People’s Hospital, No. 11 Xizhimen South Street, Xicheng District, Beijing 100044, China

**Keywords:** mitogen-activated protein kinase, vitiligo, autoimmune, melanogenesis, oxidative stress

## Abstract

Vitiligo is a chronic, acquired autoimmune disorder characterized by white skin patches resulting from the loss of epidermal melanocytes. Vitiligo may arise through multiple mechanisms, including genetic susceptibility, oxidative stress, autoimmune dysfunction, and environmental factors. Treatment strategies have focused on inhibiting melanocyte loss and stimulating repigmentation. Mitogen-activated protein kinase (MAPK) pathways regulate various cellular processes, including differentiation, survival, and inflammatory responses. The dysregulated MAPK pathways play distinct roles in the development of vitiligo through a complex interplay of melanogenesis, oxidative stress, and autoimmune responses within different cells, thereby leading to melanocyte damage. Thus, therapeutic targeting of MAPK pathways has the potential to mitigate oxidative stress-induced damage and inhibit the exaggerated autoimmunity, thereby controlling disease progression and supporting repigmentation. This review provides an overview of MAPK signaling across the multicellular network in vitiligo pathogenesis and summarizes agents that may provide new perspectives for therapeutic intervention.

## 1. Introduction

Vitiligo, an acquired depigmentation disorder, is clinically characterized by white patches or macules on the skin and has a worldwide prevalence of 0.5–2% [1]. It frequently affects exposed areas, such as the face and hands, with devastating impacts on self-esteem and self-perception [2]. The underlying mechanisms of vitiligo have not been fully elucidated. Recent advances have highlighted the key role of oxidative stress as both an initiating and a driving factor in vitiligo, leading to the loss of functional melanocytes in the epidermis [3]. Additionally, autoreactive CD8^+^ T cells attack melanocytes and contribute to further CD8^+^ T cell infiltration and immune responses, mainly through an axis involving interferon (IFN)γ, C-X-C motif chemokine ligands CXCL9 and CXCL10 [4].

The mitogen-activated protein kinase (MAPK) signaling pathway plays a significant role in melanocyte biology, including differentiation, migration and melanogenesis [5,6,7,8]. In vitiligo, the MAPK pathway is dysregulated and also involved in oxidative stress-induced destruction and autoimmune responses, eventually contributing to melanocyte death [9]. In this review, we summarize recent evidence for the role of MAPK pathways in vitiligo pathogenesis and emphasize the therapeutic potential of targeting this pathway.

## 2. Structure and Physiology of MAPK Pathways

The MAPK family of evolutionarily conserved serine/threonine protein kinases transduces diverse extracellular signals from the cell surface to the nucleus [10]. Each MAPK pathway functions through a three-tiered phosphorylation cascade, beginning with the activation of MAPK kinase kinases (MAP3Ks) that respond to various extracellular and intracellular signals and subsequently stimulate specific MAPK kinases (MAP2Ks) and MAPKs via dual phosphorylation on threonine and tyrosine residues. Once activated, MAPKs phosphorylate a variety of substrates, including transcription factors, thus dictating cellular responses and fate decisions [11].

There are three main MAPK subgroups in mammals: extracellular signal-regulated kinases (ERKs), p38 MAP kinases, and c-Jun NH2-terminal kinases (JNKs). Members of this family can be further subdivided into classic/conventional MAPKs (ERK1/2, p38, JNK, and ERK5) and atypical MAPKs (ERK3/4, ERK7, and Nemo-like kinase [NLK]) [12]. Among these, p38, ERK1/2, and JNK are the most extensively studied groups of mammalian MAPKs, and their roles in vitiligo are the primary focus of this review.

The p38 kinases, including the α, β, γ, and δ isoforms, are strongly activated by environmental stressors and inflammatory cytokines, including oxidative stress, ultraviolet (UV) irradiation, lipopolysaccharide (LPS), interleukin (IL)-1, and tumor necrosis factor alpha (TNF-α) [13]. Functionally, p38 MAPKs play a vital role in various immune and inflammatory responses by regulating the production of proinflammatory cytokines [14]. They are also involved in cell proliferation and survival, with p38α notably implicated in stress-induced cell cycle arrest and apoptosis [15,16].

There are three types of JNK—JNK1, JNK2, and JNK3—which belong to the stress-activated protein kinase (SAPK) subgroup of the MAPK family. JNK can be activated in response to various cellular stressors (e.g., heat shock, ionizing radiation, oxidative stress, DNA-damaging agents, cytokines, UV irradiation) and to a lesser extent by growth factors, certain G protein-coupled receptor ligands, and serum [11]. JNK promotes cell survival and positively regulates autophagy to counteract apoptosis [17].

ERK1/2 are the terminal kinases in the well-defined Ras/Raf/mitogen-activated protein kinase kinase (MEK)/ERK pathway, which is initially activated by growth factors (e.g., epidermal growth factor [EGF]), mitogens (e.g., protein kinase C [PKC]), and hormones with corresponding receptors [18]. ERK1/2 provide several prosurvival signals upon activation. They can block the degradation of antiapoptotic proteins (e.g., MCL1), suppress proapoptotic proteins (e.g., Bcl-2-interacting mediator of cell death [BIM]) [19], and phosphorylate Bcl-2-associated death promoter (BAD) at serine (Ser) residue 112 in human normal melanocytes, thereby contributing to cell survival [20].

In summary, p38 and JNK function as stress-sensing kinases, whereas ERK1/2 primarily transduce mitogenic and survival signals. Together, these MAPK pathways coordinately regulate vital cellular processes, including differentiation, migration, and survival/apoptosis. They are also involved in immune and inflammatory responses, playing a pathogenic role in the development of vitiligo, which is discussed in detail below.

## 3. Role of MAPK Pathways in Melanogenesis

**Figure 1:**
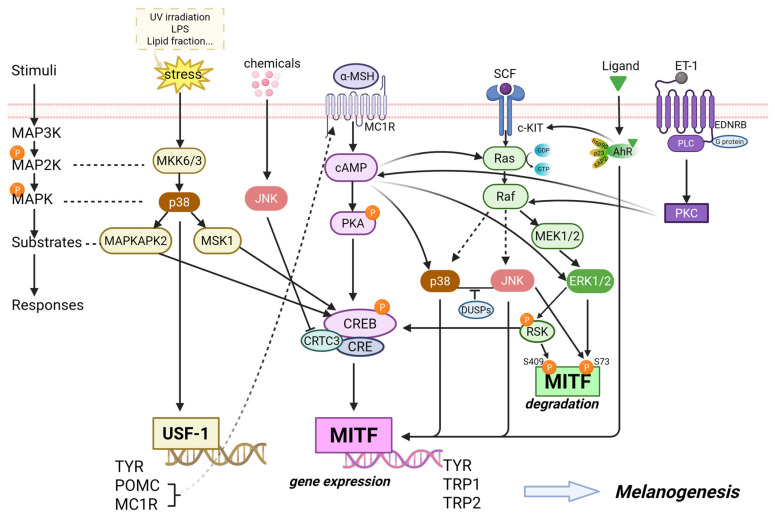
The possible mechanisms of MAPK pathway and its crosstalks with cAMP/PKA, AhR, and PKC signaling in melanogenesis. MAPK, mitogen-activated protein kinase; MAP3K, MAPK kinase kinases; MAP2K, MAPK kinases; LPS, lipopolysaccharide; USF-1, upstream stimulating factor-1; TYR, tyrosinase; POMC, pro-opiomelanocortin; MCIR, melanin cortin-1 receptor; α-MSH, a–melanocyte-stimulating hormone; c-AMP, cyclic adenosine monophosphate; PKA, c-AMP dependent protein kinase A; CREB, c-AMP response element-binding protein; CRE, c-AMP response element; CRTC3, CREB-regulated transcription coactivator 3; MITF, microphthalmia-associated transcription factor; TRP-1, tyrosinase-related protein 1; TRP-2, tyrosinase-related protein 2; SCF, stem cell factor; c-KIT, proto-oncogene c-Kit; DUSPs, dual-specificity MAPK phosphatases; RSK, ribosomal s6 kinase; AhR, aryl hydrocarbon receptor; hsp90, heat shock protein 90; p23, co-chaperone protein; XAP-2, hepatitis B virus X-associated protein 2; ET-1, Endothelin-1; EDNRB, Endothelin Receptor Type B; PLC, phospholipase C; PKC, protein kinase C. Created in BioRender. Liu, C. (2026) https://BioRender.com/np4pdbt.

Melanogenesis is a complex biochemical process primarily regulated by microphthalmia-associated transcription factor (MITF), which dictates the expression of three pigment-specific enzyme genes: tyrosinase (TYR), tyrosine-related protein-1 (TRP-1), and TRP-2 [21]. Among MAPK family members, p38 signaling enhances melanogenesis via MITF activation, while ERK1/2 exerts both inhibitory and stimulatory effects on melanin production, with JNK signaling remaining controversial (Figure 1).

Generally, p38 serves as a key driver of melanin synthesis, responding to diverse stimuli including α-melanocyte-stimulating hormone (α-MSH), UV radiation, LPS, placental lipid fractions, and electromagnetic fields [22,23,24,25,26]. Upon stress-induced activation of the MAPK kinases (MAP2Ks) MKK6 and MKK3, p38 phosphorylates downstream effectors to activate the cyclic adenosine monophosphate(cAMP) response element-binding protein (CREB). Phosphorylated CREB binds to the MITF promoter, triggering its transcription and subsequent melanin synthesis [25]. Additionally, p38 engages in a positive feedback loop with c-KIT signaling and activates upstream stimulating factor-1 (USF-1) to upregulate melanogenic genes synergistically [23,27].

The role of the JNK pathway in melanogenesis remains controversial. Activation of JNK has been linked to MITF ubiquitination and degradation, thereby downregulating melanin biosynthesis [28,29]. However, conflicting reports suggest that JNK may enhance melanogenesis in certain cellular contexts, indicating that its function may be strictly governed by the specific upstream stimuli and cellular stress levels [30,31,32]. There is evidence that α-MSH increases the expression of tyrosinase via JNK activation in melanoma cells [33]. A study revealed that flumequine promotes melanogenesis in B16-F10 cells in vitro and in zebrafish in vivo through phosphorylation of p38 and JNK [34]. A recent study demonstrated that methylsulfonylmethane (MSM) stimulates melanogenesis in Mel-Ab melanocytes by selectively activating JNK without affecting the p38 and ERK1/2 pathways, suggesting a positive regulatory function of JNK in melanin synthesis [35].

ERK1/2 signaling exhibits a dual regulatory role in melanogenesis. On the one hand, ERK1/2 can suppress melanogenesis by driving the ubiquitin proteasome-dependent degradation of MITF via downstream effectors, ribosomal s6 kinases (RSKs) [36]. This ERK-mediated degradation of MITF can be triggered by several stimuli, including endothelin-1 (ET-1) and elevated cAMP levels [37,38]. On the other hand, ERK1/2 can also promote MITF transcription through crosstalk with the cAMP/PKA pathways to indirectly phosphorylate CREB [39]. The inhibitory role of ERK1/2 signaling in melanogenesis has been well-documented in the Mel-Ab and B16-F10 cell lines [40,41,42,43,44]. ERK-mediated melanin synthesis has also been reported [45,46]. However, there remains a paucity of evidence regarding the role of ERK1/2 in melanogenesis in vitiligo melanocytes. Therefore, future investigations employing primary melanocytes directly derived from patients with vitiligo are highly warranted to elucidate the context-specific functions of the ERK1/2 pathway in the pathogenesis of this disorder.

## 4. MAPKs in Oxidative Stress-Induced Cell Damage

**Figure 2:**
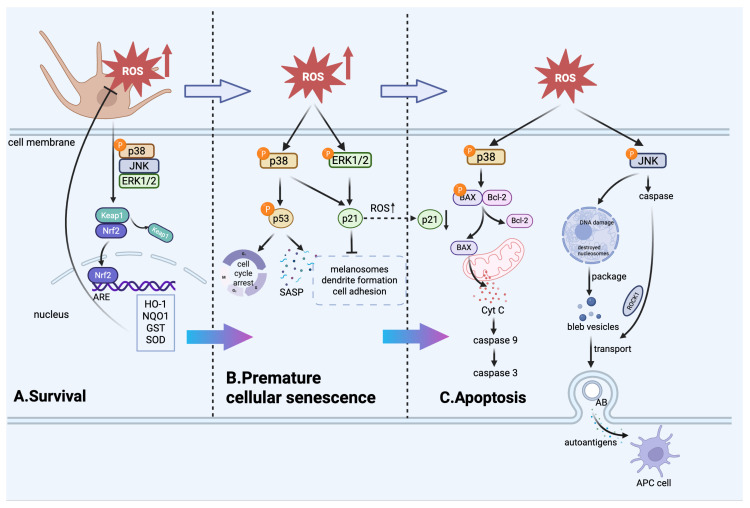
Possible role of MAPK pathway in ROS-induced melanocyte damage. (**A**) Under early or mild oxidative stress, MAPKs can protect melanocytes from ROS by activation of endogenous antioxidative defense system. (**B**) However, with long-term exposure to subcytotoxic oxidative stress, ROS induces the premature senescence of melanocytes through activation of p38 or ERK1/2. (**C**) With uncontrollable accumulation of ROS, oxidative stress eventually leads to melanocyte apoptosis through p38 or JNK. Meanwhile, activation of JNK also promotes immune destruction by mediating the presentation of autoantigens with APC cells. ROS, reactive oxygen species; Nrf2, nuclear factor erythroid 2-related factor; ARE, antioxidant responsive element; Keap1, Kelch-like ECH-associated protein 1; HO-1, heme oxygenase-1; NQO1, NAD(P)H quinone oxidoreductase 1; GST, glutathiones-transferase; SOD, superoxide dismutase; SASP, senescence-associated secretory phenotype; Cyt C, cytochrome; AB, apoptotic bodies; APC, antigen-presenting cell. Created in BioRender. Liu, C. (2026) https://BioRender.com/64bt5uu.

Oxidative stress plays a key role in the onset and progression of vitiligo. In patients with vitiligo, the epidermis is characterized by a state of oxidative stress, largely driven by excessive accumulation of H_2_O_2_. This oxidative burden has been implicated in activating signaling across the ERK1/2, JNK and p38 pathways, transmitting stress signals from the membrane to the nucleus and ultimately inducing attacks on melanocytes (Figure 2) [47].

### 4.1. MAPK-Mediated Nrf2 Antioxidant Activation

In the early stages of oxidative stress, MAPKs function as part of a protective mechanism by activating the endogenous antioxidative defense system. However, this system is often compromised in vitiligo. Activity of the Nrf2/antioxidant responsive element (ARE) pathway is reduced in vitiligo melanocytes, contributing to an imbalanced antioxidant system and uncontrollable reactive oxygen species (ROS) overload, ultimately decreasing cell viability and damaging melanocytes [48]. As important upstream regulators of Nrf2, p38, JNK, and ERK1/2 have all been shown to activate Nrf2 by prompting its phosphorylation, release from Kelch-like ECH-associated protein 1 (Keap1), and translocation into the nucleus, leading to the transcription of many antioxidant enzymes [49,50].

Several agents were used to exploit this protective axis. Aspirin and simvastatin have been shown to protect human melanocytes against H_2_O_2_-induced oxidative stress by activating Nrf2, with simvastatin exhibiting the greater antioxidative capacity of the two [49,51]. In H_2_O_2_-treated melanocytes, simvastatin potentiates ERK and JNK phosphorylation and increases p62 expression. More importantly, the reciprocal activation between ERK/JNK and p62 contributes to robust Nrf2 activation, suggesting a self-reinforcing feedback loop that strengthens the antioxidative function of Nrf2 [49]. Another study found that Cistanche deserticola polysaccharide (CDP), an effective antioxidant, likely enhances the Nrf2/HO-1 pathway through MAPK upregulation, improving melanocyte survival under oxidative stress conditions without affecting the antioxidant system under normal conditions [52].

Crucially, the protective role of MAPK is context-dependent. A related study on the JNK/Nrf2/HO-1 signaling pathway reported that JNK enhanced Nrf2 expression in normal melanocytes under ROS but reduced it in vitiligo melanocytes, suggesting that dysregulated MAPK signaling impairs the oxidative stress defense, tipping the balance toward cellular damage in vitiligo [53]. These findings underscore the complex role of MAPKs in modulating Nrf2 in melanocytes, suggesting that the capacity of MAPK signaling to enhance the antioxidative system may vary with cell status and disease course, thereby exerting either protective or proapoptotic effects.

### 4.2. MAPKs in Premature Cellular Senescence

With long-term exposure to subcytotoxic oxidative stress, melanocytes from non-lesional vitiligo skin show hyperactivated MAPK signaling, overexpression of p53, and enhanced sensitivity to proapoptotic stimuli. Reactive oxygen species (ROS)-activated p38 phosphorylates p53, leading to cell cycle arrest and reduced proliferation and expression of proteins associated with the senescence-associated secretory phenotype (SASP), including IL-6, matrix metalloproteinase-3 (MMP3), cyclooxygenase-2 (Cox-2), and insulin-like growth factor-binding protein (IGFBP)3 and IGFBP7. Driven by the SASP and dysregulation of intracellular signaling pathways, these melanocytes can influence the behavior of neighboring cells, potentially contributing to skin aging overall [54].

Premature senescence can also be induced through a p53-independent pathway. In human melanocytes, H_2_O_2_ treatment was shown to increase the phosphorylation of ERK1/2 and p38, elevating p21 expression without impacting the protein levels of p53 or p16, prompting premature senescence with impairments in melanosome formation, dendrite formation, and adhesion function. Intriguingly, with prolonged exposure to H_2_O_2_, p21 expression decreased and the number of apoptotic melanocytes increased significantly, suggesting that senescence-mediated aggravation of cell damage eventually leads to cell death [55].

Taken together, these findings characterize early-stage vitiligo as a degenerative condition, in which ROS induce premature melanocyte senescence through MAPK pathways, leading to functional impairment prior to cell death.

### 4.3. MAPKs in Melanocyte Apoptosis

As oxidative stress overwhelms antioxidant defenses, MAPK signaling shifts to drive apoptosis. The pro-oxidant state generated during melanogenesis renders melanocytes susceptible to subsequent oxidative stress, making them more vulnerable than keratinocytes and fibroblasts to its damaging effects [56]. Activation of p38 and JNK stimulates ROS-induced apoptosis in melanocytes, while ERK plays a minimal role.

Possibly the most important kinase in H_2_O_2_-induced cell death in human melanocytes [57], p38 regulates the activation of bax, bcl-2, and caspases in mitochondria-dependent apoptosis [58,59]. Triggered by oxidative stress, mitochondrial dysfunction leads to loss of transmembrane potential, calcium channel opening, and cytochrome C (Cyt C) release into the cytosol. This cascade activates caspase-9, which subsequently activates caspase-3, culminating in apoptosis [60]. Proapoptotic protein Bax promotes the release of Cyt C from mitochondria, and its activity is repressed by binding to antiapoptotic proteins such as Bcl-2 [61]. p38 activation leads to Bax phosphorylation at threonine (Thr)167, disrupting Bcl-2–Bax heterodimer formation and thus promoting apoptosis [59]. Inhibition of p38 and caspase activation lowers the release of Cyt C, the Bax/Bcl-2 ratio, and the levels of caspase-3 and caspase-9, thereby reducing H_2_O_2_-induced apoptosis in melanocytes. This suggests that p38 plays a role in regulating the mitochondria-dependent apoptosis pathway in melanocytes [9,62,63]. Consistently, antioxidants such as N-acetylcysteine (NAC), glutathione (GSH), apigenin, and (-)-epigallocatechin-3-gallate (EGCG) attenuate apoptosis via suppression of p38 [57,64,65]. Minocycline also protects melanocytes against H_2_O_2_-induced apoptosis via inhibition of JNK and p38 [66].

JNK activation promotes both apoptosis and ROS-driven immune destruction of melanocytes. ROS causes DNA demethylation via JNK phosphorylation in melanocytes, inducing overexpression of the microRNA miR-25 to increase susceptibility to oxidative stress-induced apoptosis [67]. Following the assault by ROS, autoimmune responses are initiated, leading to further melanocyte apoptosis [68]. In human vitiligo melanocytes, the JNK-related apoptosis pathway is involved in the transport of autoantigens and the formation of apoptotic bodies (ABs), serving as a bridge between oxidative stress and autoimmunity [69]. Under chronic oxidative stress, ROS accumulation activates JNK signaling, which in turn activates the caspase cascade. This leads to apoptosis, DNA damage and breakage, and nucleosome packaging into bleb-like vesicles. These vesicles migrate to the cell membrane surface and form ABs, which mediate the presentation of autoantigens to antigen-presenting cells (APCs), thereby activating adaptive immunity to intensify attacks on melanocytes [69,70].

### 4.4. MAPKs in Keratinocyte Apoptosis

**Figure 3:**
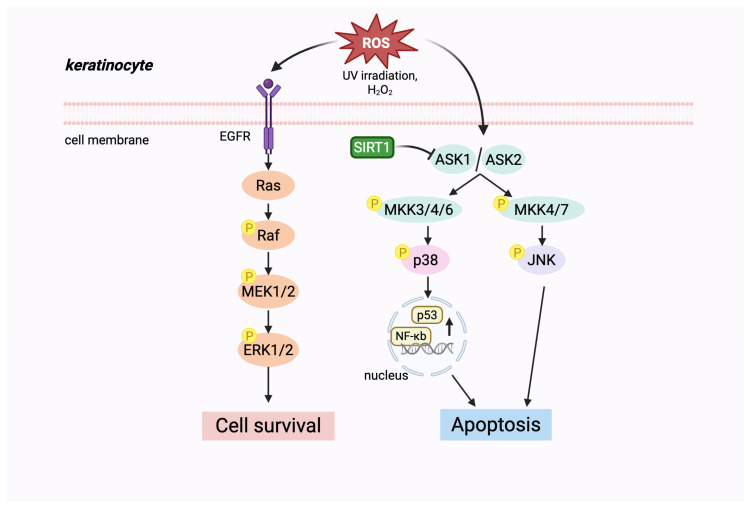
Possible role of MAPK pathway in ROS-induced keratinocyte damage. Under oxidative stress, activation of ERK1/2 promotes survival, while p38 and JNK have proapoptotic effects. EGFR, epidermal growth factor receptor; ASK, apoptosis signal–regulating kinase; NF-kB, nuclear factor kappa B. Created in BioRender. Liu, C. (2026) https://BioRender.com/lk8houe.

The actions of keratinocytes are pivotal in vitiligo, releasing and responding to a wide range of inflammatory mediators and growth factors [71]. In vitiligo lesions, the number of apoptotic keratinocytes increases with decreased SCF production and weak E-cadherin expression, leading to melanocyte detachment and apoptosis [72,73]. In cultured keratinocytes under oxidative stress, ERK1/2, p38, and JNK are all phosphorylated but exhibit distinct, independently regulated expression patterns (Figure 3) [74,75].

ERK1/2 promotes survival through the Ras/Raf/MEK pathway [54]. Following UVB irradiation, H_2_O_2_ is generated in human normal keratinocytes, stimulating phosphorylation of epidermal growth factor receptor (EGFR), followed by activation of Ras, Raf, MEK1/2, and ERK1/2 [76]. Natural antioxidants such as curcumin and capsaicin exert protective effects by enhancing ERK activation [77].

In contrast to ERK1/2, p38 and JNK exhibit proapoptotic effects. Keratinocytes from perilesional skin of patients with nonsegmental vitiligo show signs of oxidative stress and apoptosis with high levels of p38 activation, which induces apoptosis via the activities of nuclear factor kappa B (NF-kB) and p53 [77]. Apoptosis signal-regulating kinases 1 and 2 (ASK1, ASK2), upstream activators of JNK and p38 within the MAP3K family, are activated by oxidative stress and thus promote apoptosis in human keratinocytes [78]. Protective mechanisms often function by suppressing these pathways. SIRT1, a human sirtuin downregulated in skin keratinocytes exposed to UV radiation or H_2_O_2_, was found to play a protective role in perilesional vitiligo keratinocytes via Akt–ASK1. Through activation of Akt and inactivation of ASK1, SIRT1 inhibited the JNK and p38 pathways and contributed to decreased oxidative stress and apoptotic cell death [79]. A recent study suggested that Sirt1 impairment accelerates apoptosis of melanocytes under endoplasmic reticulum stress in vitiligo, but whether Sirt1 elicits its effects through MAPK pathways was not explored [80]. Furthermore, nonmetastatic melanoma protein B (GPNMB), which is specifically lost in the basal epidermal layer of vitiligo lesions and downregulated by IFN-γ, protected NHEKs from H_2_O_2_-induced oxidative stress through suppression of the JNK and p38 pathways, emerging as a treatment target [81].

The divergent roles of MAPKs in keratinocytes under oxidative stress may be attributed to the subcellular localization of ROS. Evidence suggests that cytosolic or membrane-proximal ROS, often generated in the early phase of stress, activate ERK1/2 via EGFR or dual-specificity MAPK phosphatases (DUSPs), initiating a compensatory survival response [76]. However, the activation of p38 and JNK is closely linked to the mitochondrial-derived ROS [82]. As the core pathology in vitiligo involves intrinsic mitochondrial dysfunction that generates profound oxidative stress in both melanocytes and keratinocytes, this mitochondrial stress response may override the protective ERK signals, shifting the balance toward p38/JNK dominance and ultimately precipitating keratinocyte apoptosis.

## 5. MAPKs in Autoimmune Responses of Vitiligo

**Figure 4:**
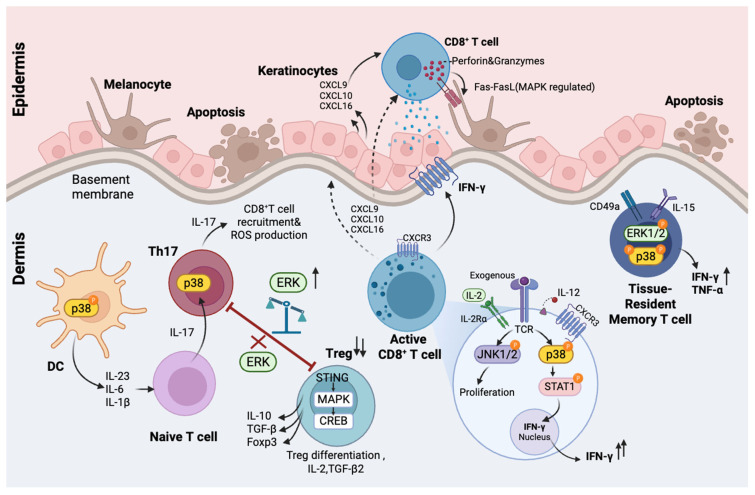
Possible mechanisms involving MAPK pathway of immune dysregulation in the pathogenesis of vitiligo. DC, dendritic cells; IL, interleukin; Th17, T helper 17; Treg, Regulatory T cell; STING, stimulator of interferon genes; TGF-β2, transforming growth factor-beta 2; TNF-α, tumor necrosis factor alpha; STAT, signal transducers and activators of transcription. Created in BioRender. Liu, C. (2026) https://BioRender.com/lzj9btn.

Abnormal immune responses are crucial in the development of vitiligo (Figure 4). It is well-established that CD8^+^ T cells are responsible for melanocyte destruction and disease progression of vitiligo. IFN-γ secreted by CD8^+^ T cells stimulates keratinocytes to produce CXCL9, CXCL10 and CXCL16, which induce initial apoptosis of melanocytes and promote CD8^+^ T cell recruitment to the epidermis, forming a positive feedback loop [83].

### 5.1. CD8^+^ T Cells

JNK can regulate the maturation and proliferation of CD8^+^ T cells through IL-2, a cytokine secreted primarily by T helper lymphocytes, and the stimulation of its receptor (IL-2R). The increased level of IL-2, both in the serum and skin biopsies of patients with vitiligo, is closely linked to CD8^+^ T cell activation and the subsequent destruction of melanocytes [84]. Interestingly, JNK1 is required for expression of IL-2Rα in CD8^+^ T cells, while JNK2 is a negative regulator of IL-2 production in CD8^+^ T cells [85].

p38 can be activated through the T cell receptor (TCR) signaling as well as cytokines (e.g., IL-12) during T cell stimulation. While p38 activation is not required for the proliferation of CD8^+^ T cells, it is required for the production of IFN-γ by CD8^+^ T cells [86]. Regulation of STAT1 transcriptional activity has been proposed as a mechanism by which p38 regulates IFN-γ transcription [87]. Additionally, activation of the p38 pathway drives the expression of the chemokine receptor CXCR3, which facilitates the migration of autoreactive CD8^+^ T cells towards vitiligo lesions via the CXCL9/CXCL10-CXCR3 axis [88].

Beyond IFN-γ signaling, CD8^+^ T cells can mediate melanocyte apoptosis by secreting perforin or granzymes and through the Fas-FasL mechanisms, which might be mediated by the MAPK pathway [89]. The specific mechanism needs further investigation in vitiligo.

### 5.2. Regulatory T Cells

Regulatory T cells (Tregs) can help terminate abnormal activation of CD8^+^ T cells and thus play a critical role in vitiligo [90]. The decreased numbers and suppressive function of Tregs contribute to unrestricted autoimmune responses, with evidence showing that the number of Tregs in the peripheral blood of patients with active vitiligo is lower than that in patients with stable vitiligo [91]. There is evidence that the activation of the stimulator of interferon genes (STING)-MAPK-CREB signaling pathway can promote Treg differentiation by inducing the expression of many cytokines, including IL-2 and transforming growth factor-beta 2 (TGF-β2), potentially combating autoimmune diseases [92].

Along with decreased Tregs, the level of T helper (Th) 17 was significantly increased in the peripheral blood of vitiligo patients. Furthermore, the imbalance of the Treg/Th17 ratio is closely related to the activity and severity of vitiligo [93]. Intriguingly, a study indicated that ERK can differentially regulate Th17 and Treg cell development in the pathogenesis of colitis. Blockade of ERK activation can inhibit Th17 cell development while upregulating Tregs with more production of IL-10, TGF-β and Foxp3 [94]. As the expression of IL-10 and Foxp3 was found to be decreased in Tregs from patients with vitiligo [95], targeting ERK may help to regulate Treg differentiation and immune disorders in vitiligo.

### 5.3. Th17 Cells

Th17 cells take part in the pathogenesis of vitiligo with increased IL-17 expression both in lesional skin and serum [96]. IL-17 attracts CD8^+^ T cells by releasing CCL20 chemokine to the peripheral areas and increases neutrophil migration, which results in ROS production, ultimately leading to melanocyte death in vitiligo [97]. The activation of p38 has been shown to be essential for the production of IL-17 by Th17 cells [98]. Meanwhile, p38 regulates the production of IL-23, IL-6, and IL-1β in dendritic cells (DCs). It has been confirmed that IL-23 and IL-6 induce pathogenic Th17 cell differentiation and IL-17 production, raising the possibility of indirect regulation of Th17 responses via DCs and p38 in vitiligo [99].

### 5.4. Tissue-Resident Memory T Cells

With 40% of patients experiencing relapses in treated areas, vitiligo recurrence is mainly mediated by CD8^+^ tissue-resident memory T (TRM) cells that exhibit cytotoxic effector functions and are dependent on IFN-γ signaling [100]. Blockade of IL-15 signaling with anti-CD122 antibodies has been shown to treat vitiligo by depleting TRM1 cells that require this cytokine for their long-term maintenance [101]. A study demonstrated that hyper-reactive CD8^+^ memory T cells exhibit significantly enhanced and prolonged phosphorylation of ERK1/2 and p38 following TCR stimulation [102]. This MAPK-driven signaling amplification may provide a mechanistic parallel for TRMs in vitiligo, whereby a lowered activation threshold with limited melanocyte-derived autoantigens allows these resident cells to produce IFN-γ and tumor necrosis factor (TNF)-α, ultimately destroying melanocytes [103].

## 6. MAPK in the Skin Microenvironment

Dermal fibroblasts in vitiligo have received considerable attention in recent years. They can modulate melanocyte activity by producing and releasing growth factors such as basic fibroblast growth factor (bFGF) and hepatocyte growth factor (HGF). bFGF activates MAPK pathways upon binding to its receptor FGFR2 on melanocytes, promoting their differentiation and proliferation [104]. bFGF also facilitates melanocyte migration through activation of the ERK and PI3K/Akt–Rac1–focal adhesion kinase (FAK)–JNK signaling pathways associated with cytoskeleton reorganization, thereby facilitating repigmentation [7]. Similarly, HGF/mesenchymal–epithelial transition factor (c-MET) signaling promotes the proliferation, survival, and motility of melanocytes by triggering the ERK and PI3K/Akt pathways [105].

Expression of bFGF in patients with vitiligo is markedly reduced in lesional skin despite being elevated in serum and blister fluid, implicating dysregulated bFGF signaling within melanocytes in the disease pathogenesis [106,107]. Interestingly, bFGF also acts in an autocrine manner on fibroblasts to accelerate their migration via PI3K/Akt–Rac1–FAK–JNK or NF-kB–JNK pathways [108,109], suggesting that MAPK dysregulation may exert broad effects in the skin microenvironment.

## 7. Synthesis of the Context-Dependent Roles of MAPKs

Throughout the pathogenesis of vitiligo, the p38, JNK, and ERK1/2 pathways do not act as static switches but rather function as dynamic, context-dependent regulators. Their ultimate biological outcomes are strictly dictated by the target cell type, the specific microenvironmental stimuli, and the temporal stage of the disease.

MAPKs can exert protective effects during the early stages of vitiligo. For instance, mild or transient oxidative stress triggers p38, JNK, and ERK1/2 to activate the Nrf2/ARE antioxidant defense system, helping melanocytes counteract ROS overload. Additionally, ERK1/2 promotes keratinocyte survival under UV irradiation, while specific activation of p38 supports compensatory melanogenesis. However, under persistent and overwhelming oxidative stress, this protective paradigm collapses. Chronic hyperactivation of p38 and JNK drives premature cellular senescence and mitochondria-dependent apoptosis in melanocytes, precipitates keratinocyte death, and facilitates the formation of apoptotic bodies that bridge oxidative stress to autoimmunity. In the immune compartment, these pathways largely orchestrate pathogenic responses, promoting the recruitment, maturation, and survival of autoreactive CD8^+^ T cells and Th17 cells and thereby perpetuating melanocyte destruction.

Recognizing these context-dependent nuances is imperative for developing targeted therapies that can selectively inhibit the pathogenic branches of MAPKs while preserving or enhancing their protective and melanogenic functions.

## 8. Therapeutic Implications and Future Perspectives

### 8.1. MAPKs in Existing Vitiligo Therapies

Topical tacrolimus (FK506), a first-line treatment for vitiligo, induces functional differentiation of immature melanoblasts by upregulating p38, PKA, and PKC. Given that phosphorylated p38 shows the most pronounced increase, this MAPK may play an important role in stimulating the differentiation and maturation of melanoblasts [5]. Perifollicular repigmentation occurs when melanoblasts become activated, differentiate, and migrate to the interfollicular epidermis and form mature melanocytes [105].

With increasing evidence highlighting the role of the Janus kinase/signal transducers and activators of transcription (JAK/STAT) pathway in the pathogenesis of vitiligo, JAK inhibitors (JAKi) have become promising therapeutic options. Topical ruxolitinib, a JAK1/2 inhibitor, was approved by the U.S. Food and Drug Administration in 2022 for the treatment of nonsegmental vitiligo [110]. Although JAK inhibitors primarily target the STAT pathway, their efficacy in vitiligo is partially mediated by the indirect suppression of MAPK signaling. For instance, inhibition of IFN-γ signaling by targeting STAT1 may downregulate IFN-γ transcription regulated by p38 in CD8^+^ T cells, leading to synergic effects on the prevention of CD8^+^ T cell recruitment [87]. Additionally, IL-15-dependent survival of CD8^+^ TRMs involves both STAT5 and ERK1/2 activation followed by JAK1/3 stimulation [111]. Thus, JAK inhibition leads to a collateral suppression of the ERK1/2 cascade, ultimately compromising TRM persistence in the skin. Investigating the crosstalk between the MAPK and JAK/STAT pathway may provide more therapeutic targets in the future.

### 8.2. Potential Treatment Targeting MAPKs

Since the p38, JNK, and ERK1/2 pathways play distinct roles in melanogenesis, the modulation of MAPKs may offer therapeutic potential for repigmentation of vitiligo. Agents that enhance melanogenesis by targeting MAPK signaling are summarized in Table 1.

Many flavonoids target the p38 pathway to exert stimulatory effects on melanin production. These include afzelin, apigenin, *Vernonia anthelmintica* (L.) Willd extract (AVE), 1,5-dicaffeoylquinic acid (1,5-dicQA), kaempferide, pinostrobin, and galangin (GA) [112,113,114,115,116,117,118]. Other natural compounds, such as methyl 3,5-di-caffeoylquinate, cannabidiol, and maclurin, have demonstrated melanogenesis activity via activation of the p38 signaling pathway in B16-F10 cells and human epidermal melanocytes [119,120,121]. Additionally, ascorbic acid, an antioxidant, accelerates p38 activation to stimulate melanogenesis in B16-F10 murine cells by enhancing expression of MITF, tyrosinase, TRP-1, and TRP-2 [122].

Novel synthetic compounds targeting MAPKs are being developed to overcome the limitations of current treatment. Traditional psoralen derivatives, such as 8-methoxypsoralen (8-MOP), used in psoralen with UVA radiation (PUVA) therapy, may carry significant side effects [123]. A newly synthesized furocoumarin derivative, 5-((diethylamino)methyl)-3-phenyl-7H-furo[3,2-g]chromen-7-one (5D3PC), showed a remarkable melanogenic effect in B16 murine cells, activating both p38 and JNK pathways. Additionally, its oral administration attenuated depigmentation in vitiligo model mice without observable toxicities, emerging as a potential drug for the repigmentation of vitiligo [30].

Several upstream agents that integrate signaling through the MAPK cascade have emerged as potential intervention points. Deficiency of aryl hydrocarbon receptor (AhR) in mice leads to reduced expression of SCF and c-KIT, critical activators of the Ras–Raf–MAPK axis [124]. A study reported that the phosphorylation levels of p38 and ERK1/2 were significantly increased by particulate matter with an equivalent diameter ≤ 2.5 µm (PM2.5) in an AhR-dependent manner both in vitro and in vivo, promoting pigmentation by regulating the expression of TYR, TYRP1, TYRP2, and MITF [125]. Taken together, these findings suggest that the SCF/c-KIT/MAPK pathway can be stimulated by AhR signaling to upregulate melanogenesis. Vitiligo epidermis exhibits significantly decreased protein expression of 5-HT7 receptor (5-HT7R). In normal human melanocytes, the 5-HT7R selective agonist LP-12 activates ERK1/2 and JNK signaling, leading to MITF upregulation and enhanced melanin formation. This mechanism may represent a novel therapeutic strategy for vitiligo, particularly in patients whose condition is exacerbated by stress [31].

Physical modulation of MAPKs offers another alternative treatment option. An in vitro study in human melanocytes showed that exposure to extremely low-frequency electromagnetic fields (EMFs) at 50 and 60 Hz could stimulate melanogenesis through activation of p38 and inhibition of phosphorylated (p)-ERK and p-JNK. EMF application may represent a therapeutic approach to induce skin repigmentation in vitiligo patients [26].

### 8.3. Translational Barriers and Future Perspectives

It is noteworthy that the vast majority of candidate agents were evaluated merely in cell lines and that some core translational barriers exist. MAPK is a highly conserved signaling pathway that plays critical roles in virtually every cell type, and MAPK signaling in vitiligo pathogenesis is not uniformly activated or suppressed. Major concerns such as cell-type targeting specificity, normal skin cell homeostasis, and off-target toxicity should be considered in clinical translation of the potential agents.

To provide a clear translational landscape, Table 2 summarizes the evidence for current therapies and emerging promising strategies that interplay with MAPK signaling. Future studies are warranted to prioritize experimental validation in cells derived from vitiligo patients, in vivo animal models and progress to clinical trials.

## 9. Conclusions

This review summarizes the multifaceted roles of MAPK pathways in the pathophysiology of vitiligo, primarily through the regulation of melanogenesis, oxidative stress-induced damage, and autoimmune responses. The dysregulated p38, ERK1/2, and JNK pathways drive disease progression through distinct mechanisms in different cell types including melanocytes, keratinocytes, dermal fibroblasts, and various immune cells.

However, the specific contributions of p38, ERK1/2, and JNK to these pathological processes remain incompletely understood. Additionally, it is crucial to identify the most amenable MAPK pathway for intervention and achieve cell-type-specific modulation. Exploring MAPK-related molecules in the serum or skin lesions as reliable biomarkers might help predict disease activity and treatment response. Further in-depth research targeting MAPK pathways may unravel the disease pathogenesis and yield new therapeutic strategies for vitiligo.

**Table 1:** Summary of agents activating MAPK pathways to induce melanogenesis as potential treatment for vitiligo.

Agent	Year	Category	Study Model	Action	Reference
THSG	2009	Natural product	B16F10 cells	Activate p38 and cAMP	[126]
Ascorbic acid	2011	Antioxidant	B16F10 cells	Activate p38	[122]
AVE	2012	Natural product	B16F10 cells and normal human melanocytes	Activate p38	[114]
3,5-diCQM	2015	Natural product	B16F10 cells and normal human melanocytes	Activate p38	[119]
Afzelin	2016	Flavonoid	Human epidermal melanocytes	Activate p38	[112]
Cannabidiol	2017	Natural product	Human epidermal melanocytes	Activate p38 and ERK1/2	[120]
Scopoletin	2017	Derivative of coumarin	B16F10 cells	Activate p38 and cAMP/PKA signaling	[127]
1,5-dicQA	2018	Natural product	B16F10 cells	Activate p38, ERK1/2 and cAMP/PKA signaling	[115]
MPFC	2018	Derivative of psoralen	B16F10 cells	Activate p38 and PKA	[128]
Maclurin	2019	A member of the benzophenone family	Human epidermal melanocytes	Activate p38 and cAMP/PKA signaling, inhibit ERK1/2	[121]
STS	2019	Natural product	B16F10 cells and zebrafish	Activate p38, JNK, ERK1/2 and cAMP/PKA signaling	[129]
Flumequine	2019	Quinolone antibiotic	B16F10 cells and zebrafish	Activate p38 and JNK	[34]
RY3-c	2019	Natural product	B16F10 cells, zebrafish, and vitiligo mouse model induced by hydroquinone	Activate p38 and ERK	[130]
Paeoniflorin	2020	Natural product	Human normal melanocytes and vitiligo mouse model treated with monobenzone; PIG1 and PIG3V	Activate ERK; activate JNK/Nrf2/HO-1	[45,53]
CDP	2020	Natural product	Human epidermal melanocytes, B16F10 cells and zebrafish	Activate p38, ERK, JNK	[52]
CWT	2021	Traditional medicine formula	B16F10 cells, vitiligo mouse model induced by hydroquinone and guinea pig model induced by hydrogen peroxide	Activate p38 and PKA	[131]
Kaempferide	2021	Flavonoid	B16F10 cells and zebrafish	Inhibit p38	[116]
Bailing tablet	2022	Chinese medicine	Network pharmacology and molecular docking	Activate p38, cAMP/PKA and PI3K/Akt signaling	[132]
Pinostrobin	2022	Flavonoid	B16F10 cells	Activate p38 and cAMP/PKA signaling	[117]
5D3PC	2022	Derivative of furocoumarin	B16F10 cells, PIG1, PIG3V and C57BL/6 mice received 5% hydroquinone	Activate p38 and JNK	[30]
Syringetin	2023	Natural product	B16F10 cells	Activate p38, JNK and PKA, inhibit ERK and PI3K/Akt signaling	[32]
MSM	2024	Chemical	Mel-Ab melanocytes	Activate JNK	[35]
4-Methylcoumarin Derivatives	2024	Derivative of coumarin	B16F10 cells	Inhibit ERK	[44]
Apigenin	2024	Flavonoid	Vitiligo mouse model induced by hydroquinone	Decrease expression of nonpho-p38	[113]
Bergaptol	2024	Metabolite of psoralen	B16F10 cells and zebrafish	Activate p38, inhibit ERK	[43]
Galangin	2025	Flavonoid	B16F10 cells and zebrafish	Activate p38 and JNK	[118]

Abbreviations: THSG, 2,3,5,4′-tetrahydroxystilbene-2-O-β-D-glucoside; AVE, *Vernonia anthelmintica* (L.) wild extract; 3,5-diCQM, Methyl 3,5-di-caffeoylquinate; 1,5-dicQA, 1,5-dicaffeoylquinic acid (1,5-dicQA); MPFC, 4-methyl-6-phenyl-2H-furo[3,2-g]chromen-2-one; STS, Sodium tanshinone IIA silate; RY3-c, 2′,3,4,4′-tetrahydrochalcone analogue; CDP, Cistanche deserticola polysaccharide; CWT, caraway tablet; 5D3PC, 5-((diethylamino)me-13 thyl)-3-phenyl-7H-furo[3,2-g]chromen-7-one); MSM, Methylsulfonylmethane.

**Table 2:** Evidence for current clinical therapies and emerging strategies interacting with MAPK signaling in vitiligo.

Therapeutic Strategy	Status/Type	MAPK-Related Mechanism	Level of Evidence/Experimental Model	Reference
Tacrolimus (FK506)	Current/Topical	Upregulates p38 to stimulate the differentiation and migration of immature melanoblasts.	Clinical	[5]
JAK inhibitors (e.g., Ruxolitinib)	Current/Topical	Collateral suppression of ERK1/2, compromising CD8+ TRM cell persistence.	Clinical	[111]
UV phototherapy	Current/Physical	Increases KIT expression via p38/CREB; activates protective ERK in keratinocytes.	Clinical	[23,79]
5-HT7R agonists (e.g., LP-12)	Emerging/Receptor target	Activates ERK1/2 and JNK to promote melanogenesis.	Preclinical/Normal human epidermal melanocytes	[31]
AhR modulators	Emerging/Receptor target	Stimulates the SCF/c-KIT/MAPK pathway to upregulate melanogenesis.	Preclinical/AhR-deficient mice and human keratinocytes	[124,125]
EMFs	Emerging/Physical	Stimulates melanogenesis via activation of p38 and inhibition of phosphorylated ERK and JNK.	Preclinical/Cultured human melanocytes and zebrafish	[26]

Abbreviations: JAK, Janus kinase; UV, ultraviolet; 5-HT7R, 5-HT7 receptor; AhR, aryl hydrocarbon receptor; EMFs, electromagnetic fields; TRM, tissue-resident memory T; SCF, stem cell factor; c-KIT, proto-oncogene c-Kit.

## Data Availability

No new data were created or analyzed in this study. Data sharing is not applicable to this article.

## References

[1] Krüger C., Schallreuter K.U. (2012). A review of the worldwide prevalence of vitiligo in children/adolescents and adults. Int. J. Dermatol..

[2] Alikhan A., Felsten L.M., Daly M., Petronic-Rosic V. (2011). Vitiligo: A comprehensive overview Part I. Introduction, epidemiology, quality of life, diagnosis, differential diagnosis, associations, histopathology, etiology, and work-up. J. Am. Acad. Dermatol..

[3] Xuan Y., Yang Y., Xiang L., Zhang C. (2022). The Role of Oxidative Stress in the Pathogenesis of Vitiligo: A Culprit for Melanocyte Death. Oxidative Med. Cell. Longev..

[4] Aulakh S., Goel S., Kaur L., Gulati S., Kaur M., Chopra D., Sarangal R., Batra J. (2025). Differential expression of serum CXCL9 and CXCL10 levels in vitiligo patients and their correlation with disease severity and stability: A cross-sectional study. Indian J. Dermatol. Venereol. Leprol..

[5] Lan C.-C.E., Wu C.-S., Chen G.-S., Yu H.-S. (2011). FK506 (tacrolimus) and endothelin combined treatment induces mobility of melanoblasts: New insights into follicular vitiligo repigmentation induced by topical tacrolimus on sun-exposed skin. Br. J. Dermatol..

[6] Gu W.-J., Ma H.-J., Zhao G., Yuan X.-Y., Zhang P., Liu W., Ma L.-J., Lei X.-B. (2014). Additive effect of heat on the UVB-induced tyrosinase activation and melanogenesis via ERK/p38/MITF pathway in human epidermal melanocytes. Arch. Dermatol. Res..

[7] Shi H., Lin B., Huang Y., Wu J., Zhang H., Lin C., Wang Z., Zhu J., Zhao Y., Fu X. (2016). Basic fibroblast growth factor promotes melanocyte migration via activating PI3K/Akt-Rac1-FAK-JNK and ERK signaling pathways. IUBMB Life.

[8] Lim J.M., Sung H.Y., Park S.W., Hwang J.S. (2025). IL-7 secreted by keratinocytes induces melanogenesis via c-kit/MAPK signaling pathway in Melan-a melanocytes. Arch. Dermatol. Res..

[9] Jin R., Hu W., Zhou M., Lin F., Xu A. (2024). Caffeic acid derivative WSY6 protects melanocytes from oxidative stress by reducing ROS production and MAPK activation. Heliyon.

[10] Ronkina N., Gaestel M. (2022). MAPK-Activated Protein Kinases: Servant or Partner?. Annu. Rev. Biochem..

[11] Cargnello M., Roux P.P. (2011). Activation and function of the MAPKs and their substrates, the MAPK-activated protein kinases. Microbiol. Mol. Biol. Rev..

[12] Coulombe P., Meloche S. (2007). Atypical mitogen-activated protein kinases: Structure, regulation and functions. Biochim. Biophys. Acta (BBA)-Mol. Cell Res..

[13] Cuadrado A., Nebreda A.R. (2010). Mechanisms and functions of p38 MAPK signalling. Biochem. J..

[14] O’Neil J.D., Ammit A.J., Clark A.R. (2018). MAPK p38 regulates inflammatory gene expression via tristetraprolin: Doing good by stealth. Int. J. Biochem. Cell Biol..

[15] Thornton T.M., Rincon M. (2009). Non-classical p38 map kinase functions: Cell cycle checkpoints and survival. Int. J. Biol. Sci..

[16] Whitaker R.H., Cook J.G. (2021). Stress Relief Techniques: P38 MAPK Determines the Balance of Cell Cycle and Apoptosis Pathways. Biomolecules.

[17] Wu Q., Wu W., Fu B., Shi L., Wang X., Kuca K. (2019). JNK signaling in cancer cell survival. Med. Res. Rev..

[18] Guo Y.-J., Pan W.-W., Liu S.-B., Shen Z.-F., Xu Y., Hu L.-L. (2020). ERK/MAPK signalling pathway and tumorigenesis. Exp. Ther. Med..

[19] Lavoie H., Gagnon J., Therrien M. (2020). ERK signalling: A master regulator of cell behaviour, life and fate. Nat. Rev. Mol. Cell Biol..

[20] Sastry K.S., Ibrahim W.N., Chouchane A.I. (2020). Multiple signaling pathways converge on proapoptotic protein BAD to promote survival of melanocytes. FASEB J..

[21] Vachtenheim J., Borovanský J. (2010). “Transcription physiology” of pigment formation in melanocytes: Central role of MITF. Exp. Dermatol..

[22] Smalley K., Eisen T. (2000). The involvement of p38 mitogen-activated protein kinase in the alpha-melanocyte stimulating hormone (alpha-MSH)-induced melanogenic and anti-proliferative effects in B16 murine melanoma cells. FEBS Lett..

[23] Corre S., Primot A., Sviderskaya E., Bennett D.C., Vaulont S., Goding C.R., Galibert M.-D. (2004). UV-induced expression of key component of the tanning process, the POMC and MC1R genes, is dependent on the p-38-activated upstream stimulating factor-1 (USF-1). J. Biol. Chem..

[24] Ahn J.H., Jin S.H., Kang H.Y. (2008). LPS induces melanogenesis through p38 MAPK activation in human melanocytes. Arch. Dermatol. Res..

[25] Saha B., Singh S.K., Mallick S., Bera R., Datta P.K., Mandal M., Roy S., Bhadra R. (2009). Sphingolipid-mediated restoration of Mitf expression and repigmentation in vivo in a mouse model of hair graying. Pigment Cell Melanoma Res..

[26] Kim Y.-M., Cho S.-E., Kim S.-C., Jang H.-J., Seo Y.-K. (2017). Effects of Extremely Low Frequency Electromagnetic Fields on Melanogenesis through p-ERK and p-SAPK/JNK Pathways in Human Melanocytes. Int. J. Mol. Sci..

[27] Mizutani Y., Hayashi N., Kawashima M., Imokawa G. (2010). A single UVB exposure increases the expression of functional KIT in human melanocytes by up-regulating MITF expression through the phosphorylation of p38/CREB. Arch. Dermatol. Res..

[28] Chung K.W., Jeong H.O., Lee E.K., Kim S.J., Chun P., Chung H.Y., Moon H.R. (2018). Evaluation of Antimelanogenic Activity and Mechanism of Galangin in Silico and in Vivo. Biol. Pharm. Bull..

[29] Kim J.-H., Hong A.-R., Kim Y.-H., Yoo H., Kang S.-W., Chang S.E., Song Y. (2020). JNK suppresses melanogenesis by interfering with CREB-regulated transcription coactivator 3-dependent MITF expression. Theranostics.

[30] Zang D., Niu C., Lu X., Aisa H.A. (2022). A Novel Furocoumarin Derivative, 5-((diethylamino)me-13 thyl)-3-phenyl-7H-furo [3,2-g] chromen-7-one Upregulates Melanin Synthesis via the Activation of cAMP/PKA and MAPKs Signal Pathway: In Vitro and In Vivo Study. Int. J. Mol. Sci..

[31] Tang H.-H., Zhang Y.-F., Yang L.-L., Hong C., Chen K.-X., Li Y.-M., Wu H. (2023). Serotonin/5-HT7 receptor provides an adaptive signal to enhance pigmentation response to environmental stressors through cAMP-PKA-MAPK, Rab27a/RhoA, and PI3K/AKT signaling pathways. FASEB J..

[32] Han H., Hyun C.G. (2023). Syringetin Promotes Melanogenesis in B16F10 Cells. Int. J. Mol. Sci..

[33] Peng H.Y., Lin C.C., Wang H.Y., Shih Y., Chou S.T. (2014). The melanogenesis alteration effects of Achillea millefolium L. essential oil and linalyl acetate: Involvement of oxidative stress and the JNK and ERK signaling pathways in melanoma cells. PLoS ONE.

[34] Karunarathne W., Molagoda I.M.N., Kim M.S., Choi Y.H., Oren M., Park E.K., Kim G.-Y. (2019). Flumequine-Mediated Upregulation of p38 MAPK and JNK Results in Melanogenesis in B16F10 Cells and Zebrafish Larvae. Biomolecules.

[35] Kim I.W., Park W.J., Yun H.Y., Kim D.S. (2024). Methylsulfonylmethane promotes melanogenesis via activation of JNK in Mel-Ab cells. Int. J. Cosmet. Sci..

[36] Jang E.J., Shin Y., Park H.J., Kim D., Jung C., Hong J.-Y., Kim S., Lee S.K. (2017). Anti-melanogenic activity of phytosphingosine via the modulation of the microphthalmia-associated transcription factor signaling pathway. J. Dermatol. Sci..

[37] Marais R., Light Y., Mason C., Paterson H., Olson M.F., Marshall C.J. (1998). Requirement of Ras-GTP-Raf complexes for activation of Raf-1 by protein kinase C. Science.

[38] Azam M.S., Kwon M., Choi J., Kim H.R. (2018). Sargaquinoic acid ameliorates hyperpigmentation through cAMP and ERK-mediated downregulation of MITF in α-MSH-stimulated B16F10 cells. Biomed. Pharmacother..

[39] Sato-Jin K., Nishimura E.K., Akasaka E., Huber W., Nakano H., Miller A., Du J., Wu M., Hanada K., Sawamura D. (2008). Epistatic connections between microphthalmia-associated transcription factor and endothelin signaling in Waardenburg syndrome and other pigmentary disorders. FASEB J..

[40] Kim D.S., Hwang E.S., Lee J.E., Kim S.Y., Kwon S.B., Park K.C. (2003). Sphingosine-1-phosphate decreases melanin synthesis via sustained ERK activation and subsequent MITF degradation. J. Cell Sci..

[41] Song Y.S., Balcos M.C., Yun H.Y., Baek K.J., Kwon N.S., Kim M.-K., Kim D.-S. (2015). ERK Activation by Fucoidan Leads to Inhibition of Melanogenesis in Mel-Ab Cells. Korean J. Physiol. Pharmacol..

[42] Cheng Z., Dai G., Hsu J., Lin J., Wu W., Su C., Wu Y. (2022). Antimelanogenesis Effect of Methyl Gallate through the Regulation of PI3K/Akt and MEK/ERK in B16F10 Melanoma Cells. Evid. Based Complement. Altern. Med..

[43] Yu X., Wang Y., Wu Z., Jia M., Xu Y., Qu H., Zhao X., Wang S., Jing L., Lou Y. (2024). Multi-technology integrated network pharmacology-based study on phytochemicals, active metabolites, and molecular mechanism of Psoraleae Fructus to promote melanogenesis. J. Ethnopharmacol..

[44] Lee Y.J., Hyun C.G. (2024). Mechanistic Insights into the Stimulatory Effect of Melanogenesis of 4-Methylcoumarin Derivatives in B16F10 Melanoma Cells. Int. J. Mol. Sci..

[45] Hu M., Chen C., Liu J., Cai L., Shao J., Chen Z., Lin L., Zheng T., Ding X., Li Z. (2020). The melanogenic effects and underlying mechanism of paeoniflorin in human melanocytes and vitiligo mice. Fitoterapia.

[46] Yanase H., Ando H., Horikawa M., Watanabe M., Mori T., Matsuda N. (2001). Possible involvement of ERK 1/2 in UVA-induced melanogenesis in cultured normal human epidermal melanocytes. Pigment Cell Res..

[47] Białczyk A., Wełniak A., Kamińska B., Czajkowski R. (2023). Oxidative Stress and Potential Antioxidant Therapies in Vitiligo: A Narrative Review. Mol. Diagn. Ther..

[48] Jian Z., Li K., Song P., Zhu G., Zhu L., Cui T., Liu B., Tang L., Wang X., Wang G. (2014). Impaired activation of the Nrf2-ARE signaling pathway undermines H_2_O_2_-induced oxidative stress response: A possible mechanism for melanocyte degeneration in vitiligo. J. Investig. Dermatol..

[49] Chang Y., Li S., Guo W., Yang Y., Zhang W., Zhang Q., He Y., Yi X., Cui T., An Y. (2017). Simvastatin Protects Human Melanocytes from H_2_O_2_-Induced Oxidative Stress by Activating Nrf2. J. Investig. Dermatol..

[50] Li X.-S., Tang X.-Y., Su W., Li X. (2020). Vitexin protects melanocytes from oxidative stress via activating MAPK-Nrf2/ARE pathway. Immunopharmacol. Immunotoxicol..

[51] Jian Z., Tang L., Yi X., Liu B., Zhang Q., Zhu G., Wang G., Gao T., Li C. (2016). Aspirin induces Nrf2-mediated transcriptional activation of haem oxygenase-1 in protection of human melanocytes from H_2_O_2_-induced oxidative stress. J. Cell. Mol. Med..

[52] Hu Y., Huang J., Li Y., Jiang L., Ouyang Y., Li Y., Yang L., Zhao X., Huang L., Xiang H. (2020). Cistanche deserticola polysaccharide induces melanogenesis in melanocytes and reduces oxidative stress via activating NRF2/HO-1 pathway. J. Cell. Mol. Med..

[53] Yuan J., Lu Y., Wang H., Feng Y., Jiang S., Gao X.-H., Qi R., Wu Y., Chen H.-D. (2020). Paeoniflorin Resists H_2_O_2_-Induced Oxidative Stress in Melanocytes by JNK/Nrf2/HO-1 Pathway. Front. Pharmacol..

[54] Bellei B., Pitisci A., Ottaviani M., Ludovici M., Cota C., Luzi F., Dell’Anna M.L., Picardo M. (2013). Vitiligo: A possible model of degenerative diseases. PLoS ONE.

[55] Hou X., Shi J., Sun L., Song L., Zhao W., Xiong X., Lu Y. (2022). The involvement of ERK1/2 and p38 MAPK in the premature senescence of melanocytes induced by H_2_O_2_ through a p53-independent p21 pathway. J. Dermatol. Sci..

[56] Koga S., Nakano M., Tero-Kubota S. (1992). Generation of superoxide during the enzymatic action of tyrosinase. Arch. Biochem. Biophys..

[57] Ning W., Wang S., Liu D., Fu L., Jin R., Xu A. (2016). Potent effects of peracetylated (-)-epigallocatechin-3-gallate against hydrogen peroxide-induced damage in human epidermal melanocytes via attenuation of oxidative stress and apoptosis. Clin. Exp. Dermatol..

[58] Wang Y., Xia C., Lun Z., Lv Y., Chen W., Li T. (2018). Crosstalk between p38 MAPK and caspase-9 regulates mitochondria-mediated apoptosis induced by tetra-α-(4-carboxyphenoxy) phthalocyanine zinc photodynamic therapy in LoVo cells. Oncol. Rep..

[59] Min H., Ghatnekar G.S., Ghatnekar A.V., You X., Bu M., Guo X., Bu S., Shen B., Huang Q. (2012). 2-Methoxyestradiol induced Bax phosphorylation and apoptosis in human retinoblastoma cells via p38 MAPK activation. Mol. Carcinog..

[60] Wu X., Yang Y., Xiang L., Zhang C. (2021). The fate of melanocyte: Mechanisms of cell death in vitiligo. Pigment Cell Melanoma Res..

[61] Wolf P., Schoeniger A., Edlich F. (2022). Pro-apoptotic complexes of BAX and BAK on the outer mitochondrial membrane. Biochim. Biophys. Acta (BBA)-Mol. Cell Res..

[62] Liu B., Jian Z., Li Q., Li K., Wang Z., Liu L., Tang L., Yi X., Wang H., Li C. (2012). Baicalein protects human melanocytes from H_2_O_2_-induced apoptosis via inhibiting mitochondria-dependent caspase activation and the p38 MAPK pathway. Free Radic. Biol. Med..

[63] Yang B., Yang Q., Yang X., Yan H.-B., Lu Q.-P. (2016). Hyperoside protects human primary melanocytes against H_2_O_2_-induced oxidative damage. Mol. Med. Rep..

[64] Park E.-S., Kim S.-Y., Na J.-I., Ryu H.-S., Youn S.-W., Kim D.-S., Yun H.-Y., Park K.-C. (2007). Glutathione prevented dopamine-induced apoptosis of melanocytes and its signaling. J. Dermatol. Sci..

[65] Lin M., Lu S.-S., Wang A.-X., Qi X.-Y., Zhao D., Wang Z.-H., Man M.-Q., Tu C.-X. (2011). Apigenin attenuates dopamine-induced apoptosis in melanocytes via oxidative stress-related p38, c-Jun NH2-terminal kinase and Akt signaling. J. Dermatol. Sci..

[66] Song X., Xu A., Pan W., Wallin B., Kivlin R., Lu S., Cao C., Bi Z., Wan Y. (2008). Minocycline protects melanocytes against H_2_O_2_-induced cell death via JNK and p38 MAPK pathways. Int. J. Mol. Med..

[67] Shi Q., Zhang W., Guo S., Jian Z., Li S., Li K., Ge R., Dai W., Wang G., Gao T. (2016). Oxidative stress-induced overexpression of miR-25: The mechanism underlying the degeneration of melanocytes in vitiligo. Cell Death Differ..

[68] Bergqvist C., Ezzedine K. (2021). Vitiligo: A focus on pathogenesis and its therapeutic implications. J. Dermatol..

[69] Tian J., Wang Y., Ding M., Zhang Y., Chi J., Wang T., Jiao B., Jian Z., Yi X., Huang Y. (2021). The Formation of Melanocyte Apoptotic Bodies in Vitiligo and the Relocation of Vitiligo Autoantigens under Oxidative Stress. Oxidative Med. Cell. Longev..

[70] Xie H., Zhou F., Liu L., Zhu G., Li Q., Li C., Gao T. (2016). Vitiligo: How do oxidative stress-induced autoantigens trigger autoimmunity?. J. Dermatol. Sci..

[71] Kovacs D., Bastonini E., Briganti S., Ottaviani M., D’Arino A., Truglio M., Sciuto L., Zaccarini M., Pacifico A., Cota C. (2022). Altered epidermal proliferation, differentiation, and lipid composition: Novel key elements in the vitiligo puzzle. Sci. Adv..

[72] Elsherif R., Mahmoud W.A., Mohamed R.R. (2022). Melanocytes and keratinocytes morphological changes in vitiligo patients. A histological, immunohistochemical and ultrastructural analysis. Ultrastruct. Pathol..

[73] Lee A.-Y., Kim N.-H., Choi W.-I., Youm Y.-H. (2005). Less keratinocyte-derived factors related to more keratinocyte apoptosis in depigmented than normally pigmented suction-blistered epidermis may cause passive melanocyte death in vitiligo. J. Investig. Dermatol..

[74] Katiyar S.K., Afaq F., Azizuddin K., Mukhtar H. (2001). Inhibition of UVB-induced oxidative stress-mediated phosphorylation of mitogen-activated protein kinase signaling pathways in cultured human epidermal keratinocytes by green tea polyphenol (−)-epigallocatechin-3-gallate. Toxicol. Appl. Pharmacol..

[75] Mantena S.K., Katiyar S.K. (2006). Grape seed proanthocyanidins inhibit UV-radiation-induced oxidative stress and activation of MAPK and NF-kappaB signaling in human epidermal keratinocytes. Free Radic. Biol. Med..

[76] Peus D., Vasa R.A., Beyerle A., Meves A., Krautmacher C., Pittelkow M.R. (1999). UVB activates ERK1/2 and p38 signaling pathways via reactive oxygen species in cultured keratinocytes. J. Investig. Dermatol..

[77] Becatti M., Prignano F., Fiorillo C., Pescitelli L., Nassi P., Lotti T., Taddei N. (2010). The involvement of Smac/DIABLO, p53, NF-kB, and MAPK pathways in apoptosis of keratinocytes from perilesional vitiligo skin: Protective effects of curcumin and capsaicin. Antioxid. Redox Signal..

[78] Trevelyan S.J., Brewster J.L., Burgess A.E., Crowther J.M., Cadell A.L., Parker B.L., Croucher D.R., Dobson R.C.J., Murphy J.M., Mace P.D. (2020). Structure-based mechanism of preferential complex formation by apoptosis signal-regulating kinases. Sci. Signal..

[79] Becatti M., Fiorillo C., Barygina V., Cecchi C., Lotti T., Prignano F., Silvestro A., Nassi P., Taddei N. (2014). SIRT1 regulates MAPK pathways in vitiligo skin: Insight into the molecular pathways of cell survival. J. Cell. Mol. Med..

[80] Zhu J., Guo Y., Luo L., Huang X., Wei T., Zuo B., Liu G., Bu W., Li C. (2025). Sirtuin1 Deficiency Could Exacerbate Melanocyte Apoptosis Under Endoplasmic Reticulum Stress. Inflammation.

[81] Nishida N., Otsu M., Mizutani Y., Ishitsuka A., Mizukami Y., Inoue S. (2025). The glycoprotein GPNMB protects against oxidative stress through enhanced PI3K/Akt signaling in epidermal keratinocytes. J. Biol. Chem..

[82] Chambers J.W., LoGrasso P.V. (2011). Mitochondrial c-Jun N-terminal kinase (JNK) signaling initiates physiological changes resulting in amplification of reactive oxygen species generation. J. Biol. Chem..

[83] Frisoli M.L., Essien K., Harris J.E. (2020). Vitiligo: Mechanisms of Pathogenesis and Treatment. Annu. Rev. Immunol..

[84] Gomes I.A., de Carvalho F.O., de Menezes A.F., Almeida F.M., Shanmugam S., de Souza Siqueira Quintans J., Quintans-Júnior L., de Moura T., Oliveira P., de Souza Araújo A.A. (2018). The role of interleukins in vitiligo: A systematic review. J. Eur. Acad. Dermatol. Venereol..

[85] Rincón M., Pedraza-Alva G. (2003). JNK and p38 MAP kinases in CD4^+^ and CD8^+^ T cells. Immunol. Rev..

[86] Merritt C., Enslen H., Diehl N., Conze D., Davis R.J., Rincón M. (2000). Activation of p38 mitogen-activated protein kinase in vivo selectively induces apoptosis of CD8^+^ but not CD4^+^ T cells. Mol. Cell. Biol..

[87] Rincón M., Enslen H., Raingeaud J., Recht M., Zapton T., Su M.S., Penix L.A., Davis R.J., Flavell R.A. (1998). Interferon-gamma expression by Th1 effector T cells mediated by the p38 MAP kinase signaling pathway. EMBO J..

[88] Guo Y.-C., Chiu Y.-H., Chen C.-P., Wang H.-S. (2018). Interleukin-1β induces CXCR3-mediated chemotaxis to promote umbilical cord mesenchymal stem cell transendothelial migration. Stem Cell Res. Ther..

[89] Chen B., Zhu L., Lin X., Kwan K.J.S., Wang J., Lu Y., Li J., Deng Y., Jiang S., Tang J. (2025). SLC4A10 impedes atherosclerosis by diminishing IFN-γ/GZMB levels of CD8(+) T cells via the MAPK pathway. Front. Immunol..

[90] Chen J., Wang X., Cui T., Ni Q., Zhang Q., Zou D., He K., Wu W., Ma J., Wang Y. (2022). Th1-like Treg in vitiligo: An incompetent regulator in immune tolerance. J. Autoimmun..

[91] Zhang Q., Cui T., Chang Y., Zhang W., Li S., He Y., Li B., Liu L., Wang G., Gao T. (2018). HO-1 regulates the function of Treg: Association with the immune intolerance in vitiligo. J. Cell. Mol. Med..

[92] Lin W., Szabo C., Liu T., Tao H., Wu X., Wu J. (2024). STING trafficking activates MAPK-CREB signaling to trigger regulatory T cell differentiation. Proc. Natl. Acad. Sci. USA.

[93] Gay-Mimbrera J., Lozano-Ojalvo D., Gómez-Arias P.J., Rivera-Ruiz I., Aguilar-Luque M., Mochón-Jiménez C., Pulido E.A., Pérez-Alegre M., Guttman-Yassky E., Ruano J. (2025). Comprehensive single-cell chromatin and transcriptomic profiling of peripheral immune cells in nonsegmental vitiligo. Br. J. Dermatol..

[94] Liu H., Yao S., Dann S.M., Qin H., Elson C.O., Cong Y. (2013). ERK differentially regulates Th17- and Treg-cell development and contributes to the pathogenesis of colitis. Eur. J. Immunol..

[95] Giri P.S., Dwivedi M., Begum R. (2020). Decreased suppression of CD8^+^ and CD4^+^ T cells by peripheral regulatory T cells in generalized vitiligo due to reduced NFATC1 and FOXP3 proteins. Exp. Dermatol..

[96] Zhen Y., Yao L., Zhong S., Song Y., Cui Y., Li S. (2016). Enhanced Th1 and Th17 responses in peripheral blood in active non-segmental vitiligo. Arch. Dermatol. Res..

[97] Beyzaee A.M., Goldust M., Patil A., Rokni G.R., Beyzaee S. (2022). The role of cytokines and vitamin D in vitiligo pathogenesis. J. Cosmet. Dermatol..

[98] Gulen M.F., Kang Z., Bulek K., Youzhong W., Kim T.W., Chen Y., Altuntas C.Z., Bak-Jensen K.S., McGeachy M.J., Do J.-S. (2010). The receptor SIGIRR suppresses Th17 cell proliferation via inhibition of the interleukin-1 receptor pathway and mTOR kinase activation. Immunity.

[99] Lee Y., Awasthi A., Yosef N., Quintana F.J., Xiao S., Peters A., Wu C., Kleinewietfeld M., Kunder S., Hafler D.A. (2012). Induction and molecular signature of pathogenic TH17 cells. Nat. Immunol..

[100] Cheuk S., Schlums H., Gallais Sérézal I., Martini E., Chiang S.C., Marquardt N., Gibbs A., Detlofsson E., Introini A., Forkel M. (2017). CD49a Expression Defines Tissue-Resident CD8^+^ T Cells Poised for Cytotoxic Function in Human Skin. Immunity.

[101] Richmond J.M., Strassner J.P., Zapata L., Garg M., Riding R.L., Refat M.A., Fan X., Azzolino V., Tovar-Garza A., Tsurushita N. (2018). Antibody blockade of IL-15 signaling has the potential to durably reverse vitiligo. Sci. Transl. Med..

[102] Schindowski K., Eckert A., Peters J., Gorriz C., Schramm U., Weinandi T., Maurer K., Frölich L., Müller W.E. (2007). Increased T-cell reactivity and elevated levels of CD8^+^ memory T-cells in Alzheimer’s disease-patients and T-cell hyporeactivity in an Alzheimer’s disease-mouse model: Implications for immunotherapy. Neuromol. Med..

[103] Boniface K., Jacquemin C., Darrigade A.S., Dessarthe B., Martins C., Boukhedouni N., Vernisse C., Grasseau A., Thiolat D., Rambert J. (2018). Vitiligo Skin Is Imprinted with Resident Memory CD8 T Cells Expressing CXCR3. J. Investig. Dermatol..

[104] Swope V.B., Medrano E.E., Smalara D., Abdel-Malek Z.A. (1995). Long-term proliferation of human melanocytes is supported by the physiologic mitogens alpha-melanotropin, endothelin-1, and basic fibroblast growth factor. Exp. Cell Res..

[105] Czyz M. (2018). HGF/c-MET Signaling in Melanocytes and Melanoma. Int. J. Mol. Sci..

[106] Seif El Nasr H., Shaker O.G., Fawzi M.M., El-Hanafi G. (2013). Basic fibroblast growth factor and tumour necrosis factor alpha in vitiligo and other hypopigmented disorders: Suggestive possible therapeutic targets. J. Eur. Acad. Dermatol. Venereol..

[107] Ozdemir M., Yillar G., Wolf R., Yillar O., Unal G., Tüzün B., Tüzün Y. (2000). Increased basic fibroblast growth factor levels in serum and blister fluid from patients with vitiligo. Acta Derm. Venereol..

[108] Shi H., Cheng Y., Ye J., Cai P., Zhang J., Li R., Yang Y., Wang Z., Zhang H., Lin C. (2015). bFGF Promotes the Migration of Human Dermal Fibroblasts under Diabetic Conditions through Reactive Oxygen Species Production via the PI3K/Akt-Rac1- JNK Pathways. Int. J. Biol. Sci..

[109] Xuan Y., Chi L., Tian H., Cai W., Sun C., Wang T., Zhou X., Shao M., Zhu Y., Niu C. (2016). The activation of the NF-κB-JNK pathway is independent of the PI3K-Rac1-JNK pathway involved in the bFGF-regulated human fibroblast cell migration. J. Dermatol. Sci..

[110] Rosmarin D., Passeron T., Pandya A.G., Grimes P., Harris J.E., Desai S.R., Lebwohl M., Ruer-Mulard M., Seneschal J., Wolkerstorfer A. (2022). Two Phase 3, Randomized, Controlled Trials of Ruxolitinib Cream for Vitiligo. N. Engl. J. Med..

[111] Mishra A., Sullivan L., Caligiuri M.A. (2014). Molecular pathways: Interleukin-15 signaling in health and in cancer. Clin. Cancer Res..

[112] Jung E., Kim J.H., Kim M.O., Jang S., Kang M., Oh S.W., Nho Y.H., Kang S.H., Kim M.H., Park S.-H. (2016). Afzelin positively regulates melanogenesis through the p38 MAPK pathway. Chem. Biol. Interact..

[113] Chauhan K., Goel F., Singh S. (2024). Apigenin protects melanocytes and improve tyrosinase activity in a hydroquinone induced vitiligo mouse model targeting P38 MAP kinase signaling: Histopathology and immunohistochemistry analysis. Naunyn Schmiedeberg’s Arch. Pharmacol..

[114] Zhou J., Shang J., Ping F., Zhao G. (2012). Alcohol extract from *Vernonia anthelmintica* (L.) willd seed enhances melanin synthesis through activation of the p38 MAPK signaling pathway in B16F10 cells and primary melanocytes. J. Ethnopharmacol..

[115] Mamat N., Lu X.Y., Kabas M., Aisa H.A. (2018). Potential anti-vitiligo properties of cynarine extracted from *Vernonia anthelmintica* (L.) Willd. Int. J. Mol. Med..

[116] Wang J., Luo L., Ding Q., Wu Z., Peng Y., Li J., Wang X., Li W., Liu G., Zhang B. (2021). Development of a Multi-Target Strategy for the Treatment of Vitiligo via Machine Learning and Network Analysis Methods. Front. Pharmacol..

[117] Yoon J.H., Youn K., Jun M. (2022). Discovery of Pinostrobin as a Melanogenic Agent in cAMP/PKA and p38 MAPK Signaling Pathway. Nutrients.

[118] Wusiman Z., Zhang A.-M., Zhang S.-S., Zhao P.-P., Kang Y.-T., Zhang Y., Li Z.-J., Huo S.-X. (2025). Galangin ameliorates PTU-induced vitiligo in zebrafish and B16F10 cells by increasing melanogenesis through activation of the p38/JNK MAPK pathway. Front. Pharmacol..

[119] Kim H.J., Kim J.S., Woo J.T., Lee I.S., Cha B.Y. (2015). Hyperpigmentation mechanism of methyl 3,5-di-caffeoylquinate through activation of p38 and MITF induction of tyrosinase. Acta Biochim. Biophys. Sin..

[120] Hwang Y.S., Kim Y.-J., Kim M.O., Kang M., Oh S.W., Nho Y.H., Park S.-H., Lee J. (2017). Cannabidiol upregulates melanogenesis through CB1 dependent pathway by activating p38 MAPK and p42/44 MAPK. Chem. Biol. Interact..

[121] Hwang Y.S., Oh S.W., Park S.-H., Lee J., Yoo J.A., Kwon K., Park S.J., Kim J., Yu E., Cho J.Y. (2019). Melanogenic Effects of Maclurin Are Mediated through the Activation of cAMP/PKA/CREB and p38 MAPK/CREB Signaling Pathways. Oxidative Med. Cell. Longev..

[122] Lee S.-A., Son Y.-O., Kook S.-H., Choi K.-C., Lee J.-C. (2011). Ascorbic acid increases the activity and synthesis of tyrosinase in B16F10 cells through activation of p38 mitogen-activated protein kinase. Arch. Dermatol. Res..

[123] Esmat S., Hegazy R.A., Shalaby S., Hu S.-C., Lan C.-E. (2017). Phototherapy and Combination Therapies for Vitiligo. Dermatol. Clin..

[124] Jux B., Kadow S., Luecke S., Rannug A., Krutmann J., Esser C. (2011). The aryl hydrocarbon receptor mediates UVB radiation-induced skin tanning. J. Investig. Dermatol..

[125] Shi Y., Zeng Z., Liu J., Pi Z., Zou P., Deng Q., Ma X., Qiao F., Xiong W., Zhou C. (2021). Particulate matter promotes hyperpigmentation via AhR/MAPK signaling activation and by increasing α-MSH paracrine levels in keratinocytes. Environ. Pollut..

[126] Jiang Z., Xu J., Long M., Tu Z., Yang G., He G. (2009). 2, 3, 5, 4′-tetrahydroxystilbene-2-O-beta-D-glucoside (THSG) induces melanogenesis in B16 cells by MAP kinase activation and tyrosinase upregulation. Life Sci..

[127] Kim D.-S., Cha S.-B., Park M.-C., Park S.-A., Kim H.-S., Woo W.-H., Mun Y.-J. (2017). Scopoletin Stimulates Melanogenesis via cAMP/PKA Pathway and Partially p38 Activation. Biol. Pharm. Bull..

[128] Yin L., Pang G., Niu C., Habasi M., Dou J., Aisa H.A. (2018). A novel psoralen derivative-MPFC enhances melanogenesis via activation of p38 MAPK and PKA signaling pathways in B16 cells. Int. J. Mol. Med..

[129] Zhong H., An X., Li Y., Cai M., Ahmad O., Shang J., Zhou J. (2019). Sodium tanshinone IIA silate increases melanin synthesis by activating the MAPK and PKA pathways and protects melanocytes from H_2_O_2_-induced oxidative stress. RSC Adv..

[130] Zhong H., Zhou J., An X.-H., Hua Y.-R., Lai Y.-F., Zhang R., Ahmad O., Zhang Y., Shang J. (2019). Natural product-based design, synthesis and biological evaluation of 2′,3,4,4′-tetrahydrochalcone analogues as antivitiligo agents. Bioorg. Chem..

[131] Abuduaini A., Lu X., Zang D., Wu T., Aisa H.A. (2021). Effects of a Traditional Caraway Formulation on Experimental Models of Vitiligo and Mechanisms of Melanogenesis. Evid. Based Complement. Altern. Med..

[132] Li J., Yang M., Song Y. (2022). Molecular mechanism of vitiligo treatment by bailing tablet based on network pharmacology and molecular docking. Medicine.

